# Multivariable prediction model for both 90-day mortality and long-term survival for individual patients with perihilar cholangiocarcinoma: does the predicted survival justify the surgical risk?

**DOI:** 10.1093/bjs/znad057

**Published:** 2023-03-15

**Authors:** Anne-Marleen van Keulen, Stefan Buettner, Joris I Erdmann, Johann Pratschke, Francesca Ratti, William R Jarnagin, Andreas A Schnitzbauer, Hauke Lang, Andrea Ruzzenente, Silvio Nadalin, Matteo Cescon, Baki Topal, Pim B Olthof, Bas Groot Koerkamp

**Affiliations:** Department of Surgery, Erasmus MC Cancer Institute, Rotterdam, the Netherlands; Department of Surgery, Erasmus MC Cancer Institute, Rotterdam, the Netherlands; Department of Surgery, Amsterdam University Medical Centres, University of Amsterdam, Amsterdam, the Netherlands; Department of Surgery, Campus Charité Mitte, Campus Virchow-Klinikum-Charité-Universitätsmedizin Berlin, Berlin, Germany; Division of Hepatobiliary Surgery, IRCCS San Raffaele Hospital, Milan, Italy; Department of Surgery, Hepatopancreatobiliary Service, Memorial Sloan Kettering Cancer Center, New York, New York, USA; Department of General, Visceral, Transplant and Thoracic Surgery, University Hospital Frankfurt, Frankfurt, Germany; Department of General, Visceral and Transplantation Surgery, University Hospital of Mainz, Mainz, Germany; Department of Surgery, Unit of Hepato-Pancreato-Biliary Surgery, University of Verona Medical School, Verona, Italy; Department of General and Transplant Surgery, University Hospital Tübingen, Tübingen, Germany; General Surgery and Transplant Unit, Department of Medical and Surgical Sciences, University of Bologna, Bologna, Italy; Department of Visceral Surgery, University Hospitals KU Leuven, Leuven, Belgium; Department of Surgery, Erasmus MC Cancer Institute, Rotterdam, the Netherlands; Department of Surgery, Amsterdam University Medical Centres, University of Amsterdam, Amsterdam, the Netherlands; Department of Surgery, Erasmus MC Cancer Institute, Rotterdam, the Netherlands

## Abstract

**Background:**

The risk of death after surgery for perihilar cholangiocarcinoma is high; nearly one in every five patients dies within 90 days after surgery. When the oncological benefit is limited, a high-risk resection may not be justified. This retrospective cohort study aimed to create two preoperative prognostic models to predict 90-day mortality and overall survival (OS) after major liver resection for perihilar cholangiocarcinoma.

**Methods:**

Separate models were built with factors known before surgery using multivariable regression analysis for 90-day mortality and OS. Patients were categorized in three groups: favourable profile for surgical resection (90-day mortality rate below 10 per cent and predicted OS more than 3 years), unfavourable profile (90-day mortality rate above 25 per cent and/or predicted OS below 1.5 years), and an intermediate group.

**Results:**

A total of 1673 patients were included. Independent risk factors for both 90-day mortality and OS included ASA grade III–IV, large tumour diameter, and right-sided hepatectomy. Additional risk factors for 90-day mortality were advanced age and preoperative cholangitis; those for long-term OS were high BMI, preoperative jaundice, Bismuth IV, and hepatic artery involvement. In total, 294 patients (17.6 per cent) had a favourable risk profile for surgery (90-day mortality rate 5.8 per cent and median OS 42 months), 271 patients (16.2 per cent) an unfavourable risk profile (90-day mortality rate 26.8 per cent and median OS 16 months), and 1108 patients (66.2 per cent) an intermediate risk profile (90-day mortality rate 12.5 per cent and median OS 27 months).

**Conclusion:**

Preoperative risk models for 90-day mortality and OS can help identify patients with resectable perihilar cholangiocarcinoma who are unlikely to benefit from surgical resection. Tailored shared decision-making is particularly essential for the large intermediate group.

## Teaser

When the oncological benefit is limited, a high-risk resection may be not be justified. Two preoperative prognostic models were created to predict 90-day mortality and survival after major liver resection for perihilar cholangiocarcinoma. These can help identify patients with resectable perihilar cholangiocarcinoma who are unlikely to benefit from surgical resection.

## Introduction

Perihilar cholangiocarcinoma (pCCA) is the most common malignancy of the biliary tree^1^. In Western countries, the incidence of pCCA is about 1–2 patients per 100 000^2,3^. The aim of surgery is resection with negative surgical margins^4,5^. Surgical resection is challenging because the tumour arises at or near the biliary confluence in proximity to the vascular inflow structures of the liver. Therefore, surgical resection typically requires major liver resection with extrahepatic bile duct resection, and often reconstruction of the portal vein and/or hepatic artery. The overall 90-day mortality rate after resection of pCCA is about 12 per cent in large nationwide Western studies^6^. This postoperative mortality ranks as one of the highest in surgical oncology. The most common cause of postoperative death is liver failure, typically owing to a small liver remnant aggravated by infectious complications^7^.

Many studies of surgery for pCCA referred to resection as the only potentially curative treatment for patients with pCCA. The chance of cure after resection, however, was only 15 per cent for patients with extrahepatic biliary tract cancer^8^. The 10-year recurrence-free survival rate after resection of pCCA was only 5 per cent^9^. Patients with lymph node metastasis rarely experience long-term survival^10^. The patient and multidisciplinary team should determine whether the predicted long-term overall survival (OS) after resection justifies the short-term surgical risk. Weighing these two outcomes requires consideration of factors for surgical risk known before operation (for example ASA fitness grade) and for OS (for example hepatic artery involvement). Several studies^11–13^ developed prognostic models for either short- or long-term postoperative outcomes. These models, however, are mostly unsuitable for preoperative decision-making because they include factors such as margin status that become available only after surgical resection.

The aim of this study was to develop two prognostic models with factors known before surgery for 90-day mortality and long-term OS after resection of pCCA. The individually predicted 90-day mortality and long-term OS can together guide the decision whether the predicted long-term OS justifies the predicted 90-day mortality.

## Methods

Patients from the collaborative multicentre international database managed by the International Hepato-Pancreato Biliary Association (IHPBA) Perihilar Cholangiocarcinoma Collaboration Group, undergoing a major liver resection for pathologically confirmed pCCA between 2000 and 2019, were included in this retrospective cohort study. A total of 25 participating centres worldwide included a median of 79 (i.q.r. 42–100) consecutive patients. Each centre collected data using a standardized and deidentified data file. Patient and tumour characteristics, clinical parameters, and laboratory results were collected retrospectively from medical archives. Patients were excluded from the present study if they had undergone minor liver resection or extrahepatic bile duct resection only, associating liver partition and portal vein ligation for staged hepatectomy, had unresectable disease at surgical exploration, R2 resection margins, or had undergone liver transplantation. Ethical approval and individual informed consent were waived by the Institutional Medical Ethics Committee of the Amsterdam University Medical Centre.

### Patient work-up and management

Variations in surgical expertise, hospital volume, and standard of care across hospitals and over time were accepted owing to the retrospective nature of the study and long inclusion period. Most patients underwent preoperative endoscopic or percutaneous transhepatic biliary drainage of the anticipated future liver remnant (FLR). When the anticipated FLR was considered inadequate, patients underwent portal vein embolization (PVE).

### Definitions and outcomes

A major liver resection was defined as resection of at least three Couinaud segments. Resection of fewer than three Couinaud segments or an extrahepatic bile duct resection only were considered minor resections. Preoperative cholangitis was defined by the presence of fever, abdominal pain, or leucocytosis requiring biliary drainage, as defined previously in the DRAINAGE trial^14^. Postoperative mortality was defined as death within 90 days after resection. OS was defined as the interval between surgery and the date of death or last follow-up. Tumour margins were considered free (R0 resection) when all resection and circumferential margins were free from tumour on pathological examination. Hepatic artery or portal vein invasion was defined by the presence of abutment of at least 180° on radiological imaging (CT and/or MRI).

### Statistical analysis

Statistical analyses were conducted with R 3.5.1 (https://cran.r-project.org). Continuous data are reported as median (i.q.r.), and were compared using Mann–Whitney *U* tests. Categorical parameters are presented as counts and percentages, with analysis using χ^2^ tests. The TRIPOD recommendations were followed for the development of this prediction model (*Supplementary material*)^15^. Multivariable models for predicting 90-day mortality were constructed using logistic regression analyses, with outcomes reported as linear predictors for comparing magnitude of correlation and ORs with corresponding 95 per cent confidence intervals. Univariable and multivariable Cox proportional hazard models were constructed for predicting OS, with outcomes reported as linear predictors for comparing magnitude of correlation and HRs with corresponding 95 per cent confidence intervals. Only factors that are available before surgery were considered for both models. Factors were included in multivariable models based on backwards selection with a cut-off of *P <* 0.050. Factors predictive of 90-day mortality were also included in the long-term survival model and vice versa. Model discrimination was presented as Harrell’s C-index. Missing data were imputed using the mice package for R 3.5.1. A sensitivity analysis was undertaken for the last 6 years of the inclusion period (2014–2019). *P* values were two-tailed and *P* < 0.050 considered to be statistically significant.

Individual patients were categorized in three groups based on arbitrary chosen cut-offs following expert consensus on the predicted outcomes of both models: favourable profile for surgical resection (90-day mortality rate below 10 per cent and predicted OS above 3 years), unfavourable profile (90-day mortality risk above 25 per cent and/or predicted OS below 1.5 years), and the intermediate group.

## Results

### Patient characteristics

Baseline characteristics of the cohort are shown in *Table 1*. In total, 2136 patients were identified in the registry; 463 patients were excluded for the following reasons: no major liver resection (227), diagnosis other than pCCA on pathological examination of the resected specimen (201), surgical resection took place before the year 2000 (21), and macroscopically unresectable disease (R2 resection, 14). This led to 1673 eligible patients in the present study. A total of 1260 patients (79.5 per cent) presented with jaundice at first presentation.

**Table 1:** Baseline characteristics of patients with pathologically confirmed perihilar cholangiocarcinoma

	No. of patients (*n* = 1673)*
Sex ratio (M : F)	962 : 711
Age (years), median (i.q.r.)	65 (56–72)
≥70	481 (33.3)
BMI (kg/m^2^), median (i.q.r.)	24.9 (22.4–27.3)
**WHO grade**	
ȃ0	371 (54.2)
ȃ1	258 (37.7)
ȃ2	51 (7.4)
ȃ3	5 (0.7)
Jaundice at presentation	1260 (79.5)
**ASA fitness grade**	
ȃI–II	942 (62.5)
ȃIII–IV	565 (37.5)
Primary sclerosing cholangitis	47 (4.0)
Preoperative biliary drainage	1389 (83.1)
Preoperative cholangitis	338 (22.3)
**Bismuth–Corlette classification**	
ȃI–III	1210 (73.5)
ȃIV	437 (26.5)
Portal vein invasion†	428 (40.3)
Hepatic artery invasion†	259 (24.5)
Tumor diameter (cm), median (i.q.r.)	2.7 (2.0–4.0)
Portal vein embolization	131 (16.1)
**Surgery**	
ȃLeft hemihepatectomy	523 (31.3)
ȃLeft extended hemihepatectomy	273 (16.3)
ȃRight Hemihepatectomy	307 (18.4)
ȃRight extended hemihepatectomy	570 (34.1)
Portal vein reconstruction	529 (33.4)
Hepatic artery reconstruction	54 (3.4)
Pancreatoduodenectomy	22 (1.5)
R1 resection margin	553 (33.3)
**AJCC T category**‡	
ȃT1–2	1052 (64.6)
ȃT3–4	577 (35.4)
**Lymph node metastases**	
ȃN0	929 (56.8)
ȃN1	663 (40.6)
ȃN2	40 (2.4)
Poor tumour differentation	376 (24.5)
**Perineural invasion**	1113 (77.0)

*Values are *n* (%) unless indicated otherwise. †Portal vein or hepatic artery invasion was defined as tumour contact of at least 180° on imaging. ‡AJCC 7th edition. T1–2: Bismuth type 1–3 without any vascular involvement.

The majority of patients underwent preoperative biliary drainage (1389, 83.0 per cent). Preoperative cholangitis occurred in 338 patients (22.3 per cent). Preoperative PVE was performed in 131 patients (16.1 per cent). Left hemihepatectomy (523, 31.3 per cent) and right extended hemihepatectomy (570, 34.1 per cent) were most commonly performed. Tumour-free margins (R0) were found in 1106 patients (66.7 per cent). Lymph node metastasis was classified as N1 in 663 patients (40.6 per cent) and N2 in 40 (2.4 per cent) (7th edition AJCC staging).

### Ninety-day mortality

The 90-day mortality rate for the entire cohort was 13.6 per cent (228 of 1673). In the group aged less than 70 years, the 90-day mortality rate was 11.2 per cent (108 of 964), compared with 18.1 per cent (87 of 481) among those aged 70 years or more. Patients who died within 90 days were more likely to have an ASA grade of III or IV (49.0 *versus* 35.8 per cent; *P* < 0.001), preoperative cholangitis (28.9 *versus* 21.3 per cent; *P* = 0.017), or a portal vein reconstruction (41.0 *versus* 32.3 per cent; *P* = 0.013). The 90-day mortality rate was the highest after extended right hemihepatectomy (105 of 570, 18.4 per cent), followed by right hemihepatectomy (51 of 307, 16.6 per cent), extended left hemihepatectomy (29 of 273, 10.6 per cent), and left hemihepatectomy (43 of 523, 8.2 per cent).

Independent risk factors for 90-day mortality were: age (OR 1.04, 95 per cent c.i. 1.03 to 1.06), ASA grade III–IV (OR 1.46, 1.08 to 1.98), preoperative cholangitis (OR 1.61, 1.15 to 2.25), tumour diameter (OR 1.03, 1.01 to 1.05), and right-sided hepatectomy (OR 1.74, 1.24 to 2.45) (*Table 2*).

**Table 2:** Univariable and multivariable logistic regression analyses of 90-day mortality

	Univariable analysis		Multivariable analysis	
	OR	*P*	OR	*P*
Male sex	1.18 (0.89, 1.58)	0.255		
Age (years)	1.04 (1.03, 1.06)	<0.001	1.04 (1.03, 1.06)	<0.001
BMI (kg/m^2^)	1.00 (0.97, 1.03)	0.985	1.01 (0.97, 1.04)	0.733
Jaundice at presentation	1.40 (0.97, 2.06)	0.079	1.14 (0.76, 1.75)	0.531
ASA grade III–IV	1.71 (1.29, 2.26)	<0.001	1.46 (1.08, 1.98)	0.014
Primary sclerosing cholangitis	0.62 (0.26, 1.28)	0.239		
Preoperative biliary drainage	1.41 (0.95, 2.15)	0.100		
Preoperative cholangitis	1.60 (1.17, 2.17)	0.003	1.61 (1.15, 2.25)	0.005
Bismuth type IV	1.19 (0.87, 1.61)	0.274	1.18 (0.85, 1.63)	0.327
Hepatic artery involvement	1.13 (0.82, 1.55)	0.452	1.23 (0.86, 1.73)	0.244
Tumour diameter (cm)	1.03 (1.01, 1.05)	<0.001	1.03 (1.01, 1.05)	0.002
Right-sided hepatectomy	2.18 (1.62, 2.94)	<0.001	1.74 (1.24, 2.45)	0.001
Extended hepatectomy	1.48 (1.12, 1.97)	0.007		
				
C-index for model			0.685	

Values in parentheses are 95% confidence intervals.

### Long-term overall survival

Median survival was 27 (95 per cent c.i. 24.6 to 28.8) months and the 5-year OS rate was 17 per cent. Median OS was 33 months after left hemihepatectomy, 27 months after left extended hemihepatectomy, 25 months after right hemihepatectomy, and 21 months after right extended hemihepatectomy. Men had worse median OS than women (25 *versus* 30 months; *P* = 0.016). Patients with worse median OS were more likely to have an ASA grade of III–IV (23 months *versus* 30 months for ASA I–II; *P* < 0.001), Bismuth IV tumours (24 months *versus* 28 months for Bismuth I–III; *P* = 0.001), hepatic artery invasion (20 *versus* 28 months; *P* < 0.001), or jaundice at presentation (24 *versus* 42 months; *P* < 0.001).

Independent poor prognostic factors for OS were: BMI (hazard ratio 1.02, 95 per cent c.i. 1.00 to1.03), jaundice at presentation (HR 1.43, 1.21 to 1.69), ASA grade III to IV (HR 1.34; 1.17 to 1.54), Bismuth type IV tumour (HR 1.20, 1.05 to 1.37), hepatic artery involvement (HR 1.27, 1.09 to 1.50), tumour diameter (HR 1.02, 1.00 to1.03), and right-sided hepatectomy (HR 1.27, 1.13 to 1.44) (*Table 3*).

**Table 3:** Univariable and multivariable survival analyses of factors associated with overall survival of patients with perihilar cholangiocarcinoma

	Univariable analysis		Multivariable analysis	
	HR	*P*	HR	*P*
Male sex	1.16 (1.03, 1.30)	0.016		
Age (years)	1.01 (1.00, 1.01)	0.025	1.01 (1.00, 1.01)	0.115
BMI (kg/m^2^)	1.02 (1.00, 1.03)	0.060	1.02 (1.00, 1.03)	0.022
Jaundice at presentation	1.52 (1.30, 1.78)	<0.001	1.43 (1.21, 1.69)	<0.001
ASA grade III–IV	1.39 (1.23, 1.58)	<0.001	1.34 (1.17, 1.54)	<0.001
Primary sclerosing cholangitis	0.95 (0.67, 1.34)	0.754		
Preoperative biliary drainage	1.21 (1.03, 1.42)	0.021		
Preoperative cholangitis	1.19 (1.02, 1.37)	0.025	1.16 (0.99, 1.36)	0.069
Bismuth type IV	1.23 (1.08, 1.41)	0.001	1.20 (1.05, 1.37)	0.009
Hepatic artery involvement	1.36 (1.14, 1.62)	<0.001	1.27 (1.09, 1.50)	0.002
Tumour diameter (cm)	1.02 (1.01, 1.03)	<0.001	1.02 (1.00, 1.03)	0.013
Right-sided hepatectomy	1.26 (1.12, 1.42)	<0.001	1.27 (1.13, 1.44)	<0.001
Extended hepatectomy	1.26 (1.12, 1.42)	<0.001		
				
C-index for model			0.601	

Values in parentheses are 95% confidence intervals.

### Long-term overall survival *versus* 90-day mortality

**Fig. 1:**
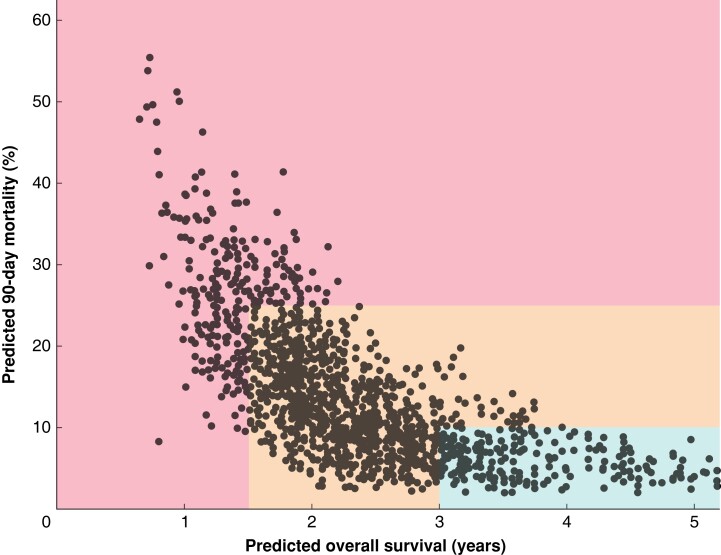
Predicted 90-day mortality *versus* predicted overall survival Patients in the green zone (favourable risk profile for surgery) all had a predicted overall survival (OS) exceeding 3 years and a predicted 90-day mortality rate below 10 per cent. Patients in the red zone (unfavourable risk profile for surgery) had a predicted OS below 1.5 years and/or a predicted 90-day mortality rate above 25 per cent. Patients in the orange zone had an intermediate risk profile. A calculator for predicting both long-term OS and 90-day mortality for the individual patient is available at https://dhoppener.shinyapps.io/risk_vs_harm_app/. The following are examples of actual patients in each zone. In the middle of the green zone was a 62-year-old woman with a BMI of 27 kg/m^2^ and ASA II. She presented with painless jaundice, and underwent endoscopic biliary drainage. She had a 4.5-cm Bismuth II perihilar cholangiocarcinoma without hepatic arterial involvement that required a left hemihepatectomy. In the middle of the orange zone was a 79-year-old woman with a BMI of 23 kg/m^2^ and ASA II. She presented with preoperative jaundice, and underwent endoscopic and percutaneous transhepatic biliary drainage. She had a 1.2-cm Bismuth II perihilar cholangiocarcinoma without hepatic arterial involvement that required a left extended hemihepatectomy. In the middle of the red zone was a 78-year-old man with a BMI of 24 kg/m^2^ and ASA IV. He presented with painless jaundice and underwent endoscopic biliary drainage. He had a 2-cm Bismuth IV perihilar cholangiocarcinoma with hepatic arterial involvement that required a right hemihepatectomy.

*Figure 1* shows both the predicted 90-day postoperative mortality rate and the predicted OS for each patient. A favourable risk profile for surgery was found for 294 patients (17.6 per cent) with a 90-day mortality rate of 5.8 per cent and median OS of 42 months; an intermediate risk profile was found for 1108 patients (66.2 per cent) with a 90-day mortality rate of 12.5 per cent and median OS of 27 months; and an unfavourable risk profile was found for 271 patients (16.2 per cent) with a 90-day mortality rate of 26.8 per cent and median OS of 16 months.

In the sensitivity analysis, HRs and ORs were comparable to those for the complete cohort, with slightly better C-index values. A calculator for predicting both long-term OS and 90-day mortality for the individual patient is available at https://dhoppener.shinyapps.io/risk_*versus*_harm_app/.

## Discussion

In this study, two preoperative prognostic models were developed for predicting 90-day mortality and long-term OS after resection of pCCA. Patients were categorized in three groups based on predicted risk for both outcomes. A favourable risk profile (90-day mortality rate below 10 per cent and predicted OS above 3 years) was observed in 17.6 per cent of patients, reflecting those who were likely to benefit from surgical resection. An unfavourable risk profile (90-day mortality rate above 25 per cent and/or predicted OS below 1.5 years) was found in 16.2 per cent of patients, who were unlikely to benefit from surgery. An intermediate risk profile, observed in 66.2 per cent of patients, would require a more tailored approach balancing patient preferences regarding surgical risk and long-term OS.

A recent systematic review^6^ reported a pooled 90-day mortality rate of 12 per cent after major liver resection for pCCA in Western countries. This is comparable to the 90-day mortality rate of 13.6 per cent in the present study, which excluded patients who underwent extrahepatic bile duct resection only. Postoperative mortality after resection for pCCA is mostly due to posthepatectomy liver failure^16,17^. Reduction in liver failure and mortality can be achieved by adequate biliary drainage and preoperative augmentation of the FLR with PVE^18^. PVE was performed in about 60 per cent of patients in a large Asian study^19^, with a low postoperative mortality rate of 2 per cent. Only 16 per cent of patients in the present study underwent PVE. More liberal use of PVE may increase the FLR volume, and decrease postoperative liver failure and mortality^20^.

Surgical resection of pCCA offers the best chance of long-term survival. The 5-year OS rate of 17 per cent and median OS of 27 months in this study is comparable to values in other series. Published median OS after resection of pCCA ranged from 20 to 40 months^21^, with 5-year OS rates ranging from 10 to 30 per cent^1^. Although long-term survival after resection of pCCA remains poor, the median OS with palliative systemic chemotherapy for advanced biliary tract cancer was only 11 months, with no survivors beyond 3 years^22^. However, these patients had advanced disease. OS in patients with resectable pCCA receiving palliative chemotherapy would probably be better. Nevertheless, the potential survival benefit of surgical resection should be carefully balanced against the risk of 90-day mortality. In addition to postoperative mortality, surgical resection is associated with considerable morbidity, with prolonged hospital stay and recovery after discharge.

The influence of (neo)adjuvant chemotherapy was not included in this preoperative risk evaluation. Only a few small retrospective studies of neoadjuvant chemotherapy for biliary tract cancer are available^23^. These studies mainly included patients with locally advanced or borderline resectable biliary tract cancer, for whom neoadjuvant chemotherapy might offer downstaging. A currently ongoing phase II trial, the NACRAC study^24^, may provide insight into whether neoadjuvant radiochemotherapy (gemcitabine + external beam radiation) can affect postoperative survival or not. The possibility of starting adjuvant chemotherapy is largely dependent on the postoperative recovery. The potential benefit of adjuvant chemotherapy is limited, as demonstrated by the BILCAP trial (capecitabine *versus* observation; HR 0.80, 95 per cent c.i. 0.63 to 1.04), the BCAT trial (gemcitabine *versus* observation; HR 1.01, 0.70 to 1.45), and the Prodige trial (gemcitabine and oxaliplatin *versus* observation; HR 1.08, 0.70 to 1.66)^25–27^.

Patients (and surgeons) might differ regarding the 90-day mortality risk that they believe would justify resection. This study assumed an arbitrary predicted 90-day mortality risk of 25 per cent above which a resection seems rarely justified. Similarly, patients (and surgeons) might also differ regarding the long-term OS that they believe would justify the surgical risks. This study assumed a predicted OS of 18 months below which a resection seems rarely justified. In surgical oncology, unfavourable patient factors and more advanced disease are often associated with both poor short- and long-term outcomes. In the present study, ASA grade III–IV, tumour diameter, and right-sided resection were associated with both worse 90-day mortality and worse long-term OS. Future research should investigate how patients and surgeons both balance surgical risk and long-term OS. Moreover, patients rely primarily on the information from the surgeon. Adequate use of shared decision-making can increase patient satisfaction and decrease decisional regret^28^.

Several staging systems for pCCA are used to guide treatment including the AJCC, Bismuth–Corlette, and Blumgart systems^29–31^. However, these staging systems consider only anatomical aspects of the tumour. To address these limitations, several prognostic models have been developed for survival after surgical resection of pCCA^12,32–35^. These models typically include margin status, nodal status, and tumour differentiation. Although these variables are strong independent prognostic factors, they are available only after surgical resection and cannot therefore be used for preoperative decision-making.

This study should be viewed in the light of several limitations. First, inherent to all retrospective studies, the diagnosis and treatment of patients differed between the 25 participating centres and changed over time during the two-decade inclusion period. However, to achieve its aim, this study required a very large cohort that was otherwise not available. Second, about one-third of patients with resectable pCCA on imaging do not undergo resection because of occult metastatic disease or more advanced disease. These patients were not included in this multicentre cohort. Therefore, the predicted OS and 90-day mortality apply only to patients who do undergo resection. Most studies presented outcomes only for patients with resected pCCA. Studies including all patients with resectable pCCA (for example the DRAINAGE and INTERCPT trials) reported a much higher perioperative mortality rate^14,36^. The proportion of patients with primary sclerosing cholangitis (PSC) was small (4 per cent), as most patients with PSC underwent liver transplantation and were therefore not included in this cohort of resections. Finally, patients with a suspected diagnosis of pCCA but a different diagnosis after resection (5–10 per cent) were not included in this multicentre cohort^37^. In the preoperative setting, the final diagnosis is not known. Consequently, observed OS will be slightly longer than predicted by the model because of patients with non-malignant disease.

## Collaborators

Perihilar Cholangiocarcinoma Collaboration Group: T.M. van Gulik, L.C. Franken, J.I. Erdmann, B.M. Zonderhuis, G. Kazemier, L.E. Nooijen (Amsterdam UMC, Amsterdam, the Netherlands); S.K. Maithel (Emory University, Atlanta, USA); J.L.A. van Vugt, J.N.M. IJzermans, R.J. Porte (Erasmus MC, Rotterdam, the Netherlands); L. Aldrighetti, R. Marino (IRCCS San Raffaele Hospital, Milan, Italy); K.J. Roberts (Queen Elizabeth Hospital, Birmingham, UK); M.C. Giglio, R. Troisi (Ghent University Hospital and Medical School, Ghent, Belgium); M. Malago, S. van Laarhoven (Royal Free Hospital, University College London, London, UK); H. Lang, F. Bartsch, R. Margies (Universitätsmedizin Mainz, Mainz, Germany); R. Alikhanov, M. Efanov (Moscow Clinical Scientific Center, Moscow, Russia); A. Guglielmi, (University School of Medicine of Verona, Verona, Italy); H.Z. Malik, L.M. Quinn (University Hospital Aintree, Liverpool, UK); C. Gomez-Gavara, C. Dopazo (all D'Hebron University Hospital, Barcelona, Spain); E. de Savornin Lohman, P. de Reuver (Radboud University Medical Center, Nijmegen, the Netherlands); S.W.M. Olde Damink, S. Bouwense (Maastrischt University Medical Center, Maastricht, the Netherlands); E. Sparrelid, H. Jansson, S. Glig (Karolinska University Hospital, Stockholm, Sweden); M. Schmelzle, C. Benzing (Charité - Universitätsmedizin Berlin, Berlin, Germany); M. Serenari, M. Ravaioli (l'Università di Bologna, Bologna, Italy); J. Rolinger, I. Capobianco (Tübingen University Hospital, Tübingen, Germany); E. Schadde, J. Heil (Cantonal Hospital Winterthur, Zurich, Switserland); Q.I. Molenaar, J. Hagendoorn (University Medical Center Utrecht, Utrecht, the Netherlands); W.O.Bechstein, T.A. Nguyen (Universitätsklinikum Frankfurt, Frankfurt, Germany); J. Geers (University Hospital KU Leuven, Leuven, Belgium); P. Lodge, A. Hakeem, R. Prasad (St. James's University Hospital, Leeds, UK); J. Bednarsch (University Hospital RWTH Aachen, Aachen, Germany); F. Hoogwater, M.A. de Boer (University Medical Center Groningen, Groningen, the Netherlands).

## Supplementary Material

znad057_Supplementary_Data

## Data Availability

Data were obtained from the Perihilar Cholangiocarcinoma Collaboration Group. Data are available from the corresponding author on reasonable request.
