# Supplementary figures and images for: Multivariable prediction model for both 90-day mortality and long-term survival for individual patients with perihilar cholangiocarcinoma: does the predicted survival justify the surgical risk?
Source: Br J Surg. 2023 Mar 15;110(5):599–605. doi: 10.1093/bjs/znad057 (PMC10364519; doi:10.1093/bjs/znad057)

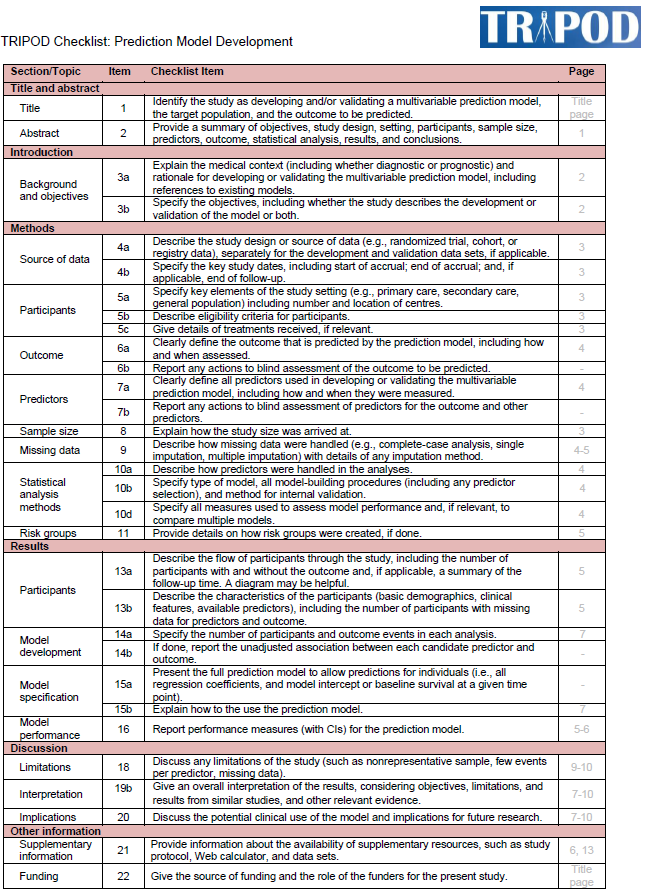

Supplement: znad057_Supplementary_Data [file znad057_supplementary_data.docx]
